# The association between insulin resistance and the consumption of nut including peanut, pine nut and almonds in working-aged Korean population

**DOI:** 10.1017/S1368980021003803

**Published:** 2022-07

**Authors:** Sung Keun Park, Chang-Mo Oh, Ju Young Jung

**Affiliations:** 1 Center for Cohort Studies, Total Healthcare Center, Kangbuk Samsung Hospital, Sungkyunkwan University, School of Medicine, Seoul, The Republic of Korea; 2 Department of Preventive Medicine, School of Medicine, Kyung Hee University, Seoul, The Republic of Korea; 3 Total Healthcare Center, Kangbuk Samsung Hospital, Sungkyunkwan University, School of Medicine, Seoul, The Republic of Korea

**Keywords:** Nutrition, Nut, Insulin sensitivity, Insulin resistance

## Abstract

**Objective:**

Studies have reported that nuts intake is potentially beneficial to cardiometabolic health. However, there have been heterogeneous results regarding the association between nut intake/consumption and the risk of diabetes mellitus (DM). Insulin resistance (IR) is a major pathophysiology of DM. Thus, this study was to assess the association between nuts consumption and IR.

**Design:**

A retrospective cross-sectional study.

**Setting:**

Multivariable-adjusted OR and 95 % CI for increased IR (adjusted OR (95 % CI)) were calculated according to the frequency of consuming one serving dose (15 g) of nuts including peanut, pine nut and almond (< 1/month, 1/month–1/week, 1–3/week, 3–5/week, ≥ 5/week). Elevated IR was defined in homoeostasis model assessment-insulin resistance corresponding to the fourth quartile levels within each study group. Subgroup analysis was conducted for gender, glycaemic status (normal, prediabetes and DM) and age (≥ and < 40 years).

**Participants:**

379 310 Koreans who received health check-up.

**Results:**

Compared with nut consumption < 1/month (reference), nuts consumption ≥ 1/month had the lower OR and 95 % CI for elevated IR (1/month–1/week: 0·90 (95 % CI 0·89, 0·92), 1–3/week: 0·90 (95 % CI 0·87, 0·92), 3–5/week: 0·94 (95 % CI 0·89, 0·98) and ≥ 5/week: 0·90 (95 % CI 0·86, 0·94)). This association was more remarkable in women, normal glycaemic group and young age group (< 40 years). However, men, prediabetes, DM and old age group did not show the significant association.

**Conclusion:**

Nuts consumption ≥ 1/month was less associated with elevated IR. Increased nuts consumption may have a favourable effect on IR.

Good dietary habits are an essential part in preventing non-communicable diseases and promoting health^(1)^. Nuts are used as healthy food rich in good materials including dietary fibre, vegetable protein, minerals, phytosterols and phenolic compounds^(2)^. Studies have suggested that nuts consumption may be helpful in preventing cardiometabolic diseases^(3–5)^. Increased nuts consumption was associated with decreased risk of CVD^(3)^, diabetes mellitus (DM)^(4)^, obesity and metabolic syndrome^(5)^. Additionally, a recent study showed that nuts consumption was inversely associated with all-cause and cause-specific mortality in the 50 045 participants of the Golestan Cohort Study^(6)^. Previous studies have shown the potential mechanism for the favourable effect of nut consumption on cardiometabolic health^(7,8)^. A meta-analysis demonstrated that nut consumption had anti-inflammatory effect leading to improvements in inflammatory markers including C-reactive protein, cytokines like IL-6 and IL-10^(7)^. Additionally, increased nuts consumption was significantly associated with the low levels of total cholesterol and LDL-cholesterol in normal and hyperlipidaemic individuals^(8)^.

Insulin resistance (IR) refers to a pathological condition in which cells fail to respond normally to insulin. IR is characterised by the increase of insulin demand and subsequent hyperinsulinaemia by pancreatic compensation^(9)^. IR is a driving factor for DM and metabolic syndrome^(10)^, being strongly associated with atherosclerotic CVD. Interventional studies have demonstrated that increased nuts consumption was potentially effective in improving IR through beneficial glycaemic response^(11–13)^. However, there was a conflicting result regarding the association between nuts consumption and IR^(14)^. Additionally, meta-analyses did not show the consistent results about the preventive effect of nuts consumption against DM^(4,15)^. These results warrant the necessity of further studies to investigate the association of nuts consumption with IR.

To better understand the influence of nuts consumption on IR, we quantified the association with increased IR according to the frequency of nut consumption with specific one serving amount in working-aged Korean population. Additionally, subgroup analysis was conducted for gender, glycaemic status and age, which was to clarify the association in given characteristics of population.

## Methods

### Study design and participants

The present study data were obtained from Kangbuk Samsung Health Study. Kangbuk Samsung Health Study is a cohort study to investigate the medical data of Koreans who have received medical health check-up in Kangbuk Samsung Hospital. Korea’s Industrial Safety and Health law regulates that all of Korean employees should receive medical health check-up annually or biennially. According to this law, Korean companies make contract with hospitals to make their employees and their spouse receive health check-up. Among study participants in Kangbuk Samsung Health Study, we initially enrolled 441 476 subjects who had responded to semi-quantitative FFQ between March 2011 and December 2018. Out of them, 62 166 subjects with missing values in FFQ or other covariates were excluded. Finally, the total number of eligible study participants was 379 310.

### Clinical and laboratory measurements

Study data included medical history assessed by self-administered questionnaire, anthropometric measurements and laboratory measurements. All study subjects were asked to respond to a health-related behaviour questionnaire, which included the topics of alcohol consumption, smoking and exercise. The degree of physical activity was evaluated by the Korean-validated version of the International Physical Activity Questionnaire short form validated by a previous study^(16)^. Detailed description for the methods of anthropometric and laboratory measurements is included in the previous study of our group^(17)^.

Glycaemic status was classified into normal glycaemia, prediabetes and DM. DM was defined as one of the following conditions: fasting glucose ≥ 126 mg/dl, HbA1c ≥ 6·5 % and a prior diagnosis of DM^(18)^. Fasting glucose of 100–125 mg/dl or HbA1c of 5·7–6·5 % was regarded as prediabetes. IR was evaluated by calculating homoeostasis model assessment-insulin resistance (HOMA-IR) as following formula: HOMA-IR = fasting serum insulin (pmol/l) × fasting serum glucose (mmol/l)/22·5^(19)^. When all of study participants were categorised by quartile levels of HOMA-IR, subjects corresponding to the fourth quartile of HOMA-IR were regarded as group with increased IR state.

Blood samples for laboratory measurements were drawn from an antecubital vein after more than 12 h’ fasting on the day of health check-up. The Laboratory Medicine Department at Kangbuk Samsung Hospital has been accredited by the Korean Society of Laboratory Medicine and the Korean Association of Quality Assurance for Clinical Laboratories. The laboratory also participates in the survey proficiency testing provided by the College of American Pathologists.

### FFQ and nuts consumption assessments

Dietary intake was assessed by semi-quantitative FFQ developed based on Korean National Health and Nutrition Examination Survey^(20)^. The reliability and validity of semi-quantitative FFQ were demonstrated in studies from the Korea Centers for Disease Control and Prevention^(21)^.

In FFQ, food consumption frequency was composed of nine categories (i.e. never or rarely, once a month, two or three times a month, once or twice a week, three or four times a week, five or six times a week, once a day, twice a day and three times a day) and three serving sizes for each food based on the sixth edition of the Korean Food Composition table^(22)^. The serving size of nuts was categorised into 7·5 g (half serving size), 15 g (one serving size) and 22·5 g (one and half serving size) in the sixth edition of the Korean Food Composition table^(23)^. Food photographs with usual intake portions also were included to enhance subjects’ understanding and study reliability (online supplementary material, Supplemental Fig. 1)

All subjects were asked about their intake of peanut, pine nut and almonds, which were categorised as nuts. One serving dose of nuts was 15 g, which was used in classifying the frequency of nut consumption into five groups as follows: rare (< 1 serving/month), 1/month–1/week (1 serving/month ≤ and < 1 serving/week), 1–3/week (1 serving/week ≤ and < 3 serving/week), 3–5/week (3 serving/week ≤ and < 5 serving/week) and frequent (≥ 5 serving/week). Total energy intake and nutrient intakes were calculated using the nutrient database of the Korean Nutrient Society (CAN-pro 3·0, Computer Aided Analysis Program 3·0 for professional, Korean Society of Nutrition), which was based on the sixth edition of the Korean Food Composition table^(22)^. All nutrients were total-energy adjusted using the residual method^(23)^. The validity and reproducibility of the FFQ were previously evaluated in a study for 124 Korean subjects^(21)^. In that study, two surveys of FFQ were consecutively performed with 1-year interval. The median of correlations between the two surveys of FFQ was 0·45 for all nutrient intakes and 0·39 for nutrient densities.

## Statistical analyses

Study participants were assigned into five study groups based on the frequency of nuts consumption (rare, 1/month–1/week, 1–3/week, 3–5/week and frequent).

Within five study groups, data are presented as means ± standard deviation for continuous variables and as proportions for categorical variables. The linear regression model was used for continuous variables, and Cochran–Armitage trend test was used for categorical variable to assess linear response between nut consumption and biochemical, health-related behaviour, chronic disease.

Unadjusted and multivariable-adjusted logistic regression analysis was used in calculating the OR and 95 % CI for increased IR (adjusted OR (95 % CI)) across five study groups. As mentioned above, increased IR was defined in subject corresponding to the fourth quartile level of HOMA-IR. After checking for multicollinearity, selected variables were enrolled into adjusting covariates of multivariable logistic regression analysis. (selected model: age, regular exercise, BMI, smoking, alcohol intake (g/d), TAG, DM, hypertension, sex, total energy intake). The goodness of fit for logistic regression model was evaluated by Hosmer–Lemeshow test. Subgroup analysis was conducted for gender, glycaemic status (normal glycaemia, prediabetes and DM) and age (≥ and < 40 years). Each of sex, DM and age was excluded from adjusting covariates in subgroup analysis for gender, glycaemic status and age.

All statistical analyses were performed using R 3.6.3 (R Foundation for Statistical Computing), and a value of *P* < 0·05 (two-sided) was considered statistically significant in all analyses.

## Result

The main clinical characteristics of study participants are presented in Table 1. Most of study participants were working-aged Koreans with mean age (sd) of 38·7 ± 9·1 years, and only 3·3 % (*n* 12 357) of study participants were older than 60 years. The average nuts consumption was 12·7 g/week or approximately one serving/week. Compared with rare consumption group (< 1 serving/month), frequent consumption group (≥5 serving/week) had the higher levels in age, fasting glucose, physical activity, the prevalence of DM and hypertension, and nutritional intake in total calorie, carbohydrate, protein and fat. However, HOMA-IR, the proportion of elevated IR and fasting insulin level were lower in frequent consumption group than rare consumption group.

**Table 1:** Baseline characteristics of the study participants stratified by the frequency of nuts consumption

Nuts consumption	< 1/month (*n* 187 390)		1/month–1/week (*n* 115 832)		1–3/week (*n* 44 223)		3–5/week (*n* 14 980)		≥5/week (*n* 16 885)		*P* for trend
	Mean	SD	Mean	SD	Mean	SD	Mean	SD	Mean	SD	
Men											
* n*	100 219		67 515		24 659		7202		8558		<0·001
* *%	53·5 %		58·3 %		55·8 %		48·1 %		50·7 %		
Age (year)	37·9	8·8	39·1	8·8	39·2	9·4	41·2	10·9	40·8	10·5	<0·001
Fasting glucose (mmol/l)	5·27	0·79	5·29	0·80	5·27	0·84	5·28	0·82	5·28	0·89	<0·001
Fasting insulin (pmol/l)	44·45	29·86	42·36	29·17	43·06	28·47	41·67	29·86	41·67	28·47	<0·001
HOMA-IR	1·53	1·22	1·48	1·16	1·51	1·20	1·47	1·28	1·46	1·22	<0·001
HbA1c	5·5	0·5	5·6	0·5	5·6	0·5	5·6	0·5	5·6	0·5	<0·001
TAG (mmol/l)	1·27	0·91	1·27	0·87	1·23	0·86	1·16	0·77	1·17	0·82	<0·001
BMI (kg/m^2^)	23·2	3·4	23·4	3·3	23·6	3·4	23·3	3·3	23·5	3·5	<0·001
Average alcohol use (g/d)	15·1	23·7	14·5	22·4	14·0	22·9	12·1	21·2	13·2	23·0	<0·001
Current smoking											
* *%	21·4 %		20·9 %		18·6 %		13·6 %		16·2 %		<0·001
High physical activity											
* *%	14·3 %		17·0 %		20·0 %		23·2 %		25·8 %		<0·001
Diabetes mellitus											
* *%	3·5 %		4·0 %		4·0 %		4·9 %		5·1 %		<0·001
Hypertension											
* *%	10·4 %		11·3 %		11·7 %		13·4 %		13·0 %		<0·001
Total energy intake (kJ/d)	5676·9	2699·9	6465·5	2730·5	6777·2	2989·9	6756·3	3321·7	7612·4	4052·2	<0·001
Carbohydrate intake (g/d)	227·5	109·1	254·4	110·6	257·8	115·5	255·6	122·5	276·0	144·2	<0·001
Protein intake (g/d)	46·0	25·5	54·6	26·1	59·5	30·0	59·9	35·5	69·4	43·6	<0·001
Fat intake (g/d)	27·4	19·6	32·8	19·4	37·8	23·0	38·6	26·5	48·9	34·1	<0·001
Nut consumption (g/d)	0·0	0·1	0·9	0·5	3·8	0·8	7·5	0·0	16·2	8·6	<0·001
Increased IR (≥1·86)											
* n*	48 334		28 244		10 822		3499		3922		<0·001
* *%	25·8 %		24·4 %		24·5 %		23·4 %		23·2 %		

HOMA-IR, homoeostasis model assessment-insulin resistance; IR, insulin resistance.

Continuous variables are expressed as mean (± sd), and categorical variables are expressed as number (percentage (%)).

The frequency of nuts consumption: < 1 serving/month, 1/month–1/week (1 serving/month ≤ and < 1 serving/week), 1–3/week (1 serving/week ≤ and < 3 serving/week), 3–5/week (3 serving/week ≤ and < 5 serving/week), ≥ 5 serving/week.

Table 2 shows the unadjusted and the multivariable-adjusted OR and 95 % CI for elevated IR according to the frequency of nuts consumption. Compared with rare consumption, more nuts consumption had the lower adjusted OR and 95 % CI for elevated IR (rare consumption: 1·00 (reference), 1/month–1/week: 0·90 (95 % CI 0·89, 0·92), 1–3/week: 0·90 (95 % CI 0·87, 0·92), 3–5/week: 0·94 (95 % CI 0·89, 0·98) and frequent: 0·90 (95 % CI 0·86, 0·94)). This association was more clearly identified in women (rare consumption: 1·00 (reference), 1/month–1/week: 0·90 (95 % CI 0·87, 0·93), 1–3/week: 0·87 (95 % CI 0·83, 0·90), 3–5/week: 0·89 (95 % CI 0·84, 0·95) and frequent: 0·78 (95 % CI 0·73, 0·83)).

**Table 2:** The OR and 95 % CI for increased insulin resistance according to the frequency of nuts consumption

Serving	< 1/month	1/month–1/week	1–3/week	3–5/week	≥ 5/week
All participant					
* n*	187 390	115 832	44 223	14 980	16 885
Unadjusted OR	1·00	0·93	0·93	0·88	0·87
95 % CI	Reference	0·91, 0·94	0·91, 0·95	0·84, 0·91	0·84, 0·90
Multivariable-adjusted OR	1·00	0·90	0·90	0·94	0·90
95 % CI	Reference	0·89, 0·92	0·87, 0·92	0·89, 0·98	0·86, 0·94
Increased IR (≥1·86)					
* n*	48 334	28 244	10 822	3499	3922
* *%	25·8 %	24·4 %	24·5 %	23·4 %	23·2 %
Men					
* n*	100 219	67 515	24 659	7202	8558
Unadjusted OR	1·00	0·92	0·98	0·96	1·01
95 % CI	Reference	0·90, 0·94	0·95, 1·02	0·90, 1·01	0·96, 1·07
Multivariable-adjusted OR	1·00	0·92	0·93	0·99	0·99
95 % CI	Reference	0·89, 0·94	0·90, 0·97	0·92, 1·05	0·93, 1·05
Increased IR (≥2·03)					
* n*	25 591	16 240	6220	1778	2207
* *%	25·5 %	24·1 %	25·2 %	24·7 %	25·8 %
Women					
* n*	87 171	48 317	19 564	7778	8327
Unadjusted OR	1·00	0·87	0·84	0·84	0·72
95 % CI	Reference	0·85, 0·89	0·81, 0·87	0·80, 0·89	0·68, 0·76
Multivariable-adjusted OR	1·00	0·90	0·87	0·89	0·78
95 % CI	Reference	0·87, 0·93	0·83, 0·90	0·84, 0·95	0·73, 0·83
Increased IR (≥1·66)					
* n*	23 137	11 573	4551	1813	1712
* *%	26·5 %	24·0 %	23·3 %	23·3 %	20·6 %

IR, insulin resistance.

Adjusted for age, regular exercise, BMI, smoking, alcohol intake (g/d), TAG, diabetes mellitus, hypertension, sex, total energy intake (sex excluded in gender subgroup).

The frequency of nuts consumption: < 1 serving/month, 1/month–1/week (1 serving/month ≤ and < 1 serving/week), 1–3/week (1 serving/week ≤ and < 3 serving/week), 3–5/week (3 serving/week ≤ and < 5 serving/week), ≥ 5 serving/week.

Subgroup analyses for glycaemic status are presented in Table 3. Normal glycaemia showed that the nuts consumption ≥ 1 serving/month was less associated with elevated IR than rare consumption (1/month–1/week: 0·90 (95 % CI 0·88, 0·93), 1–3/week: 0·87 (95 % CI 0·84, 0·90), 3–5/week: 0·89 (95 % CI 0·84, 0·94) and frequent: 0·87 (95 % CI 0·82, 0·92)). However, this association was not observed in both prediabetes and DM.

**Table 3:** The OR and 95 % CI for increased insulin resistance according to the frequency of nuts consumption in glycaemic subgroups (normal glycaemia, prediabetes and diabetes mellitus)

Serving	<1/month	1/month–1/week	1–3/week	3–5/week	≥5/week
Normal glycaemia					
* n*	112 915	66 430	26 283	8678	9732
Unadjusted OR	1·00	0·90	0·89	0·82	0·82
95 % CI	Reference	0·88, 0·92	0·86, 0·91	0·78, 0·86	0·78, 0·86
Multivariable-adjusted OR	1·00	0·90	0·87	0·89	0·87
95 % CI	Reference	0·88, 0·93	0·84, 0·90	0·84, 0·94	0·82, 0·92
Increased IR (≥ 1·58)					
* n*	29 548	16 039	6279	1952	2188
* *%	26·2 %	26·2 %	23·9 %	22·5 %	22·5 %
Prediabetes					
* n*	67 968	44 793	16 157	5563	6295
Unadjusted OR	1·00	0·88	0·94	0·84	0·84
95 % CI	Reference	0·86, 0·90	0·90, 0·98	0·79, 0·90	0·79, 0·89
Multivariable-adjusted OR	1·00	0·91	0·95	1·02	0·99
95 % CI	Reference	0·88, 0·94	0·91, 1·00	0·95, 1·10	0·92, 1·06
Increased IR (≥ 2·18)					
* n*	17 786	10 652	4035	1279	1441
* *%	26·2 %	23·8 %	25·0 %	23·0 %	22·9 %
Diabetes mellitus					
* n*	6507	4609	1783	739	858
Unadjusted OR	1·00	0·83	0·93	0·75	0·75
95 % CI	Reference	0·76, 0·91	0·83, 1·05	0·62, 0·90	0·63, 0·89
Multivariable-adjusted OR	1·00	0·90	1·01	0·98	0·94
95 % CI	Reference	0·82, 1·00	0·88, 1·17	0·79, 1·22	0·76, 1·15
Increased IR (≥ 3·97)					
* n*	1746	1080	454	159	185
* *%	26·8 %	23·4 %	25·5 %	21·5 %	21·6 %

IR, insulin resistance.

Adjusted for age, regular exercise, BMI, smoking, alcohol intake (g/d), TAG, hypertension, sex, total energy intake.

The frequency of nuts consumption: < 1 serving/month, 1/month–1/week (1 serving/month ≤ and < 1 serving/week), 1–3/week (1 serving/week ≤ and < 3 serving/week), 3–5/week (3 serving/week ≤ and < 5 serving/week), ≥ 5 serving/week.

In age subgroup analysis (Table 4), group with age ≥ 40 years did not show the significant association between the frequency of nuts consumption and elevated IR. In contrast, group with age < 40 years showed the lower association of nuts consumption ≥ 1 serving/month with elevated IR, compared with rare consumption.

**Table 4:** The OR with 95 % CI for increased insulin resistance according to the frequency of nuts consumption in age subgroups

Serving	<1/month	1/month–1/week	1–3/week	3–5/week	≥5/week
Age ≥ 40					
* n*	63 405	45 233	17 283	6864	7542
Unadjusted OR	1·00	0·94	0·98	0·90	0·920
95 % CI	Reference	0·91, 0·97	0 94, 1 02	0 85, 0 95	0 87, 0 97
Multivariable-adjusted OR	1·00	0·95	1·02	1·01	1·02
95 % CI	Reference	0·92, 0·98	0·98, 1·07	0·94, 1·08	0·96, 1·09
Increased IR (≥ 1·90)					
* n*	16 236	11 055	4349	1624	1816
* *%	25·6 %	24·4 %	25·2 %	23·7 %	24·1 %
Age < 40					
* n*	123 985	70 599	26 940	8116	9343
Unadjusted OR	1·00	0·91	0·90	0·84	0·81
95 % CI	Reference	0·89, 0·93	0·87, 0·93	0·80, 0·89	0·77, 0·85
Multivariable-adjusted OR	1·00	0·88	0·84	0·87	0·80
95 % CI	Reference	0·86, 0·91	0·81, 0·87	0·82, 0·93	0·75, 0·85
Increased IR (≥ 1·84)					
* n*	32 239	17 098	6485	1850	2073
* *%	26·0 %	24·2 %	24·1 %	22·8 %	22·2 %

IR, insulin resistance.

Adjusted for age, regular exercise, BMI, smoking, alcohol intake (g/d), TAG, diabetes mellitus, hypertension, sex, total energy intake.

The frequency of nuts consumption: < 1 serving/month, 1/month–1/week (1 serving/month ≤ and < 1 serving/week), 1–3/week (1 serving/week ≤ and < 3 serving/week), 3–5/week (3 serving/week ≤ and < 5 serving/week), ≥ 5 serving/week.

Online supplementary material, Supplemental Table 1 shows the mean levels of HOMA-IR across the frequency of nuts consumption in each subgroup. Cut-offs for the quartile level of HOMA-IR in each study group are presented in online supplementary material, Supplemental Table 2 (all participants: 1·86, men: 2·03, women: 1·66, normal glycaemic group: 1·58, prediabetes: 2·18, DM: 3·97, age ≥ 40: 1·90 and age < 40: 1·84).

## Discussion

In analysis for working-aged Koreans, increase in nuts consumption more than one serving dose per month is less associated with elevated IR, compared with rare nuts consumption. In particular, frequent nuts consumption more than 5 times/week had the lowest mean levels of HOMA-IR and the lowest association with elevated IR. These results suggest that increased nuts consumption is potentially beneficial in improving IR. Previous studies have published reports in line with ours.

In an interventional study for nine healthy volunteers, the addition of almonds to white bread resulted in a progressive reduction in the glycaemic index of the composite meal in a dose-dependent manner^(11)^. When pistachios nut was co-consumed with carbohydrate meal in ten healthy volunteers, pistachio nut attenuated the relative glycaemic response of carbohydrate meal^(12)^. Additionally, in an analysis for 16 784 study participants from the National Health and Nutrition Examination Survey between 2005 and 2010, multivariate (age, sex, energy intake and race) adjusted mean of glucose/insulin homoeostasis parameters and TAG-glucose index decreased with the increase in quartile of nuts intake^(23)^. However, the number of study subjects in interventional studies was less than tens, which limits the generalisation of findings. Results from National Health and Nutrition Examination Survey did not include BMI in adjusting covariates, and thus, did not show whether the effect of nuts on DM is independent of BMI.

In contrast, our results were obtained from analysis for 379 310 subjects with adjustment for covariates including BMI. Thus, it is likely that our study shows the more generalised findings than previous works.

Although previous studies have demonstrated that nuts consumption is associated with favourable glycaemic response and improvement in IR^(11–13)^, it is still debatable whether increased nuts consumption could decrease the risk of DM. Moreover, epidemiological results have suggested the gender difference regarding the effect of nuts consumption on the risk of DM. In the Nurses’ Health Study for 83 818 women, Jiang *et al.* showed nuts consumption was associated with a 29 % decreased risk of incident DM^(24)^. In particular, nuts consumption ≥ 5 times/week reduced the risk of DM by 45 % in women with BMI < 25 kg/m^2^. Another analysis for Nurses’ Health Study indicated that walnut consumption ≥ 2 servings/week was significantly associated with 24 % risk reduction of DM even after adjusting covariates including BMI^(25)^. On the contrary to the analysis for women, prospective cohort study for 20 224 male participants of the Physicians’ Health Study did not show the statistically significant association between nuts consumption and DM was found in either lean or overweight/obese participants^(26)^. In our analysis, the association between increased nuts consumption and decreased HOMA-IR was observed only in women. Our result may be an explanation for the gender difference for the effect of nuts consumption on DM. The favourable effect of nuts on insulin sensitivity and glycaemic response is stronger in women than men, which may result in the decreased risk of DM only in women. Difference of the hormonal milieu between men and women may partly account for the findings of gender difference. It is known that oestrogen has the protective effect against metabolic dysfunction. In particular, oestrogen has the favourable effect on glucose homoeostasis via promoting glucose uptake in muscle and suppressing glucose production in liver^(27,28)^. The reduction of oestrogen in postmenopausal women accelerates the development of IR and DM^(29)^. Considering the mean age of our study participants (38·7 ± 9·1 years), the most of our female subjects might be the premenopausal with the sufficient production of oestrogen. Therefore, the favourable effect of oestrogen might enhance the association between nuts consumption and HOMA-IR in women.

In the present study, we conducted subgroup analysis by glycaemic status to identify the potential impact of nuts consumption on the improvement of IR in prediabetic and diabetic population. Our results indicate that increased nuts consumption is not significantly associated with the lower probability of IR in subjects with prediabetes and DM. Recent meta-analysis also showed that the daily intake of 56 g tree nut did not make significant treatment effect for fasting insulin and HOMA-IR in subjects with DM^(30)^. Several hypotheses can be raised to explain our finding. In diabetic patients, it is plausible that the effect of diabetic medication on IR surpasses that of nuts consumption. Additionally, it is recognised that the amount of nuts consumption in our study subjects was relatively lower than that in the Western population (56 g/d). One serving size of nuts consumption was 15 g in our study, and group with most frequent consumption was more than five serving sizes per a week. Thus, the lower intake of nuts is less effective in improving IR in diabetic patients. Lastly, there was a possibility that people with prediabetes or DM are likely to consume more nuts to promote health. It is widely believed that nuts are beneficial in enhancing cardiometabolic health. In practice, our results indicated that frequent nuts consumption had the higher levels in fasting glucose and the proportion of DM than rare consumption. This perplexing finding may be explained by a good dietary habit of subjects with DM or prediabetes. However, it seems that nuts intake is insufficient to restore insulin sensitivity in diabetic or prediabetic patients. Our subgroup analysis by glycaemic status indicated that only normal glycaemic group had the significant association between elevated nuts consumption and IR. In this context, it is inferred that nuts consumption should be recommended even in subjects with normal glycaemia as the purpose of preventing DM.

In our age subgroup analysis, younger age group (< 40 years) showed the significant association between increased nuts consumption and decreased HOMA-IR, whereas older age group (≥ 40 years) did not show the significant association. This finding indicates that the favourable effect of nuts consumption on IR was more remarkable in the young age than the older age. Therefore, it is noted that the mean age of study participants was over 40 years in previous works failing to show the significant association between nuts consumption and the risk of DM^(15,26)^. Nuts consumption in the older age may be less effective to restore IR and derangement of glucose homoeostasis, compared with that in the younger age group. In this regard, our finding emphasises the importance of nuts consumption in the young age group.

The strengths of our study are large sample size and well-recruited anthropometric and laboratory measurements. These merits allow us to evaluate the association with elevated IR according to the frequency of nuts consumption in the various subgroups.

Nonetheless, the limitation of the study should be considered.

First, cross-sectional design of study limits the inference for the causative relationship between nuts consumption and IR. Second, nuts intake in FFQ was not validated. Although a study validated reliability and reproducibility for FFQ^(21)^, each item in FFQ has not been validated. Third, one serving dose of nuts (15 g) used in our analysis is relatively smaller than that (28 g) commonly used in other studies^(4,15,24–26)^. Therefore, it is less likely that our findings are generalised into other ethnic and regional groups. Fourth, our study subjects were non-random convenience sample derived from one hospital. Therefore, our study subjects were non-representative for general Korean population with a possibility of selection bias.

In conclusion, we found that nuts consumption ≥ one serving dose (15 g) per month was less associated with elevated IR. This association was more prominently observed in women, normal glycaemic group and young age group. These findings may provide the novel insight regarding the influence of nuts consumption on glucose homoeostasis and IR.

## Supplementary material

For supplementary material accompanying this paper visit https://doi.org/10.1017/S1368980021003803. click here to view supplementary material
