# Supplementary material for: The association between insulin resistance and the consumption of nut including peanut, pine nut and almonds in working-aged Korean population
Source: Public Health Nutr. 2021 Sep 6;25(7):1904–11. doi: 10.1017/S1368980021003803 (PMC9991658; doi:10.1017/S1368980021003803)
Supplement: Supplementary file 1 [file S1368980021003803sup.zip › S1368980021003803sup001.pptx]

## Slide 1
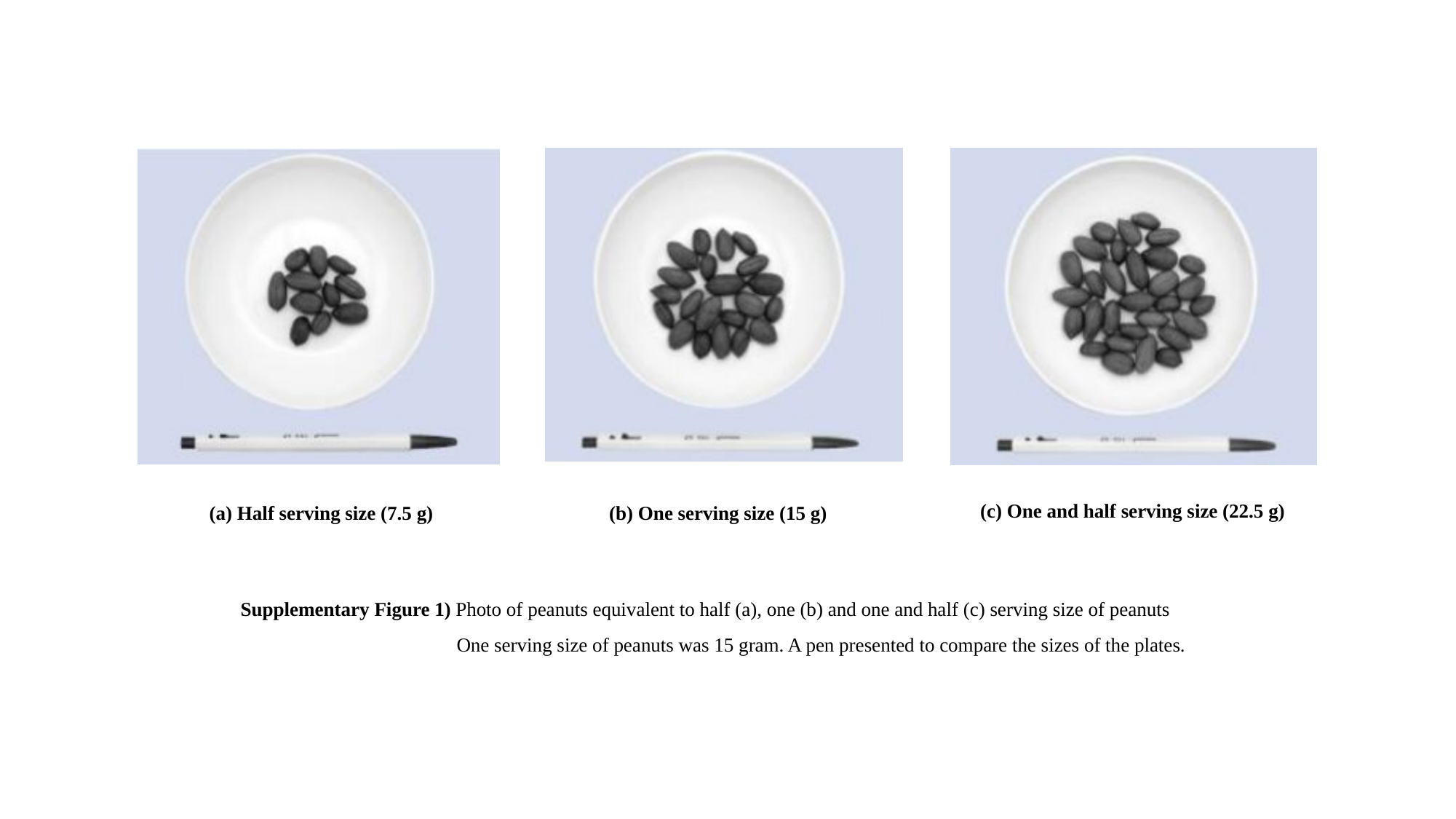

(c) One and half serving size (22.5 g)
(a) Half serving size (7.5 g)
(b) One serving size (15 g)
Supplementary Figure 1) Photo of peanuts equivalent to half (a), one (b) and one and half (c) serving size of peanuts
 One serving size of peanuts was 15 gram. A pen presented to compare the sizes of the plates.
