# Supplementary material for: The association between insulin resistance and the consumption of nut including peanut, pine nut and almonds in working-aged Korean population
Source: Public Health Nutr. 2021 Sep 6;25(7):1904–11. doi: 10.1017/S1368980021003803 (PMC9991658; doi:10.1017/S1368980021003803)
Supplement: Supplementary file 1 [file S1368980021003803sup.zip › S1368980021003803sup002.docx]

**Supplementary Table 1.** The mean value of HOMA-IR (±SD) according to the frequency of nuts consumption in each study group

| **Serving** | < 1/month | 1/month – 1/week | 1-3/week | 3-5/week | ≥5/week | P for trend |
| --- | --- | --- | --- | --- | --- | --- |
| Men | 1.66 ± 1.28 | 1.60 ± 1.24 | 1.66 ± 1.32 | 1.62 ± 1.31 | 1.67 ± 1.38 | 0.098 |
| Women | 1.39 ± 1.13 | 1.32 ± 1.05 | 1.31 ± 0.99 | 1.32 ± 1.24 | 1.24 ± 0.99 | <0.001 |
| Normal glycemia | 1.28 ± 0.80 | 1.23 ± 0.83 | 1.24 ± 0.78 | 1.21 ± 0.74 | 1.19 ± 0.74 | <0.001 |
| Prediabetes | 1.79 ± 1.15 | 1.69 ± 1.13 | 1.75 ± 1.20 | 1.66 ± 1.17 | 1.67 ± 1.15 | <0.001 |
| Diabetes mellitus | 3.33 ± 3.21 | 3.08 ± 2.84 | 3.20 ± 3.08 | 3.01 ± 3.64 | 2.95 ± 3.16 | <0.001 |
| Age ≥ 40 years | 1.55 ± 1.27 | 1.52 ± 1.16 | 1.56 ± 1.35 | 1.50 ± 1.30 | 1.51 ± 1.36 | <0.001 |
| Age < 40 years | 1.52 ± 1.20 | 1.47 ± 1.18 | 1.47 ± 1.10 | 1.44 ± 1.27 | 1.41 ± 1.10 | <0.001 |

HOMA-IR: homeostasis model assessment-insulin resistance, SD: standard deviation

**Supplementary Table 2.** The Range of HOMA-IR in each study group

| **Quartile** | Q1 | Q2 | Q3 | Q4 |
| --- | --- | --- | --- | --- |
| All participants | 0.03 – 0.80 | 0.81 – 1.23 | 1.24 – 1.85 | 1.86 – 88.4 |
| Men | 0.04 – 0.87 | 0.88 – 1.34 | 1.35 – 2.02 | 2.03 – 88.4 |
| Women | 0.03 – 0.74 | 0.75 – 1.12 | 1.13 – 1.65 | 1.66 – 87.5 |
| Normal glycemia | 0.03 – 0.72 | 0.73 – 1.08 | 1.09 – 1.57 | 1.58 – 88.4 |
| Prediabetes | 0.03 – 0.96 | 0.97 – 1.46 | 1.47 – 2.17 | 2.18 – 53.1 |
| Diabetes mellitus | 0.06 – 1.45 | 1.46 – 2.43 | 2.44 – 3.96 | 3.97 – 87.5 |
| Age ≥ 40 | 0.03 – 0.80 | 0.81 – 1.24 | 1.25 – 1.89 | 1.90 – 69.4 |
| Age < 40 | 0.03 - 0.81 | 0.82 – 1.23 | 1.24 – 1.83 | 1.84 – 88.4 |

HOMA-IR: homeostasis model assessment-insulin resistance
